# The Distribution of Coronal Plane Alignment of the Knee Classification in a Sample of Spanish Southeast Osteoarthritic Population: A Retrospective Cross-Sectional Observational Study

**DOI:** 10.3390/medicina60101612

**Published:** 2024-10-02

**Authors:** Vicente J. León-Muñoz, José Hurtado-Avilés, Mirian López-López, Fernando Santonja-Medina, Joaquín Moya-Angeler

**Affiliations:** 1 Department of Orthopaedic Surgery and Traumatology, Hospital General Universitario Reina Sofía, Avda. Intendente Jorge Palacios, 1, 30003 Murcia, Spain; jmoyaangeler@gmail.com; 2 Instituto de Cirugía Avanzada de la Rodilla (ICAR), C. Barítono Marcos Redondo 1, 30005 Murcia, Spain; 3 Department of Surgery, Paediatrics and Obstetrics & Gynaecology, Faculty of Medicine, Avda. Buenavista 32, 30120 Murcia, Spain; fernando@santonjatrauma.es; 4 Sports & Musculoskeletal System Research Group (RAQUIS), University of Murcia, Avda. Buenavista 32, 30120 Murcia, Spain; joseaviles@um.es; 5 Servicio de Coordinación y Aplicaciones Informáticas, Subdirección General de Tecnologías de la Información (Servicio Murciano de Salud), C. Central, 7, 30100 Murcia, Spain; mirindalopez@gmail.com; 6 Department of Orthopaedic Surgery and Traumatology, Hospital Clínico Universitario Virgen de la Arrixaca, Ctra. Madrid-Cartagena, s/n, 30120 Murcia, Spain

**Keywords:** knee replacement arthroplasty, total knee arthroplasty, osteoarthritis, coronal plane alignment of the knee (CPAK), knee phenotypes, kinematic alignment, mechanical alignment

## Abstract

*Background and Objectives:* The Coronal Plane Alignment of the Knee (CPAK) classification is a pragmatic distribution of nine phenotypes for coronal knee alignment that can be used on healthy and arthritic knees. Our study aimed to describe the CPAK distributions in a Spanish southeast osteoarthritic population and compare them to other populations’ published alignment distributions. *Method and Materials:* Full-leg standing X-rays of the lower limb from 528 cases originating from the so-called Vega Alta del Segura (southeast of the Iberian Peninsula) were retrospectively analysed. We measured the mechanical hip–knee–ankle, lateral distal femoral, and medial proximal tibial angles. We calculated the arithmetic hip–knee–ankle angle and the joint line obliquity to classify each case according to the criteria of the CPAK classification. *Results:* Based on the aHKA result, 59.1% of the cases were varus (less than −2°), 32.7% were neutral (0° ± 2°), and 8.2% were valgus (greater than +2°). Based on the JLO result, 56.7% of the cases had a distal apex (less than 177°), 39.9% had a neutral apex (180° ± 3°), and 3.4% had a proximal apex (greater than 183°). The most common CPAK distribution in our Spanish southeast osteoarthritic population was type I (30.7%), followed by type IV (25.9%), type II (21%), type V (11.2%), type III (5%), type VI (2.8%), type VII (2.4%), type VIII (0.6%), and type IX (0.4%). *Conclusions:* We described the distribution according to the CPAK classification in a sample of the osteoarthritic population from southeastern Spain. In our sample, more than 75% of the patients were classified as type I, II, and IV.

## 1. Introduction

The Coronal Plane Alignment of the Knee (CPAK) classification is a pragmatic distribution of nine phenotypes for coronal knee alignment based on constitutional limb alignment and joint line obliquity that can be used on healthy and arthritic knees [1]. The CPAK is not the only way to classify knee morphology in the coronal plane. However, the CPAK classification is perhaps most widely used by knee surgeons.

Despite the progress of our understanding, there are several controversial aspects of total knee arthroplasty (TKA) surgery. One aspect that has not yet been clarified is optimal alignment in TKA [2,3,4,5,6]. In 1973, Michael Freeman postulated the basis of mechanical alignment (MA) with osteotomies perpendicular to the femoral and tibial mechanical axes in the coronal and sagittal planes [7]. Until a few years ago, we assumed that restoring a neutral mechanical axis increased implant survival [8]. However, MA does not consider the significant variability of knee anatomy in different individuals and causes deviations from the native constitutional alignment of the limb and the obliquity of the joint line, as well as alterations in the physiological laxity of the knee ligament complexes. The functional outcomes of the mechanical-aligned TKA are inconsistent, and discomfort during activities in daily life can be a significant cause of patient dissatisfaction after mechanical-aligned TKA [9,10]. The concepts of constitutional varus [11] and kinematic alignment (KA) [12] were introduced more than a decade ago, and we are progressively witnessing a proliferation of articles published on different individualised alignment techniques in TKA surgery [13,14,15,16,17,18]. These individualised alignment philosophies in TKA surgery are based on several recent studies describing limb alignment in non-osteoarthritic (OA) and OA populations [11,19,20,21,22].

MacDessi et al. have proposed a system for categorising nine knee phenotypes, called the Coronal Plane Alignment of the Knee (CPAK) classification [1]. This system evaluates two criteria: constitutional limb alignment (or arithmetic hip–knee–ankle angle (aHKA angle)) and joint line obliquity (JLO). These criteria can be calculated according to the mechanical lateral distal femoral angle (LDFA) and the mechanical medial proximal tibial angle (MPTA). Constitutional limb alignment is described as varus, neutral, or valgus. The aHKA is calculated by subtracting the LDFA value from the MPTA value. The JLO is described as apex distal, neutral, or proximal, and is calculated by adding the value of the MPTA to the value of the LDFA. Three subgroups of aHKA are crossed with the three subgroups of JLO to yield the nine CPAK types [23]. The CPAK classification system is standardised, simple, and universal. It is progressively being used to assess the difference in the phenotypic distribution of alignment in different populations [1,24,25,26,27,28,29,30,31], and we already have information on geographical differences in CPAK types in healthy and arthritic knees [23].

To the best of our knowledge, no phenotypic analysis of the Spanish population using the CPAK classification has been performed. Therefore, this study aimed to describe the CPAK distributions in a Spanish southeast OA population and compare them to the published alignment distribution in populations from different geographical areas.

## 2. Materials and Methods

This study was retrospective, cross-sectional, and observational. Data from 528 consecutive cases in 468 OA patients who underwent primary TKA performed by a single senior surgeon were retrospectively analysed. The initial cohort comprised 300 women (64%) and 168 men (36%). All the cases originated from the so-called Vega Alta del Segura, a region of Murcia, an autonomous community of Spain located in the southeast of the Iberian Peninsula. All participants’ standard preoperative full-leg standing X-rays of the lower limb (LLRs) were evaluated. In all cases, the patients were to undergo TKA surgery, and, therefore, had Kellgren–Lawrence grade 3 or 4 knees. We excluded cases that, due to previous interventions (e.g., osteotomies) or fractures, presented an alteration of the native constitutional axes. To avoid possible axes alterations, we also excluded patients with hip replacements [32,33].

Following the same methodology as in other papers published by our group [34,35], we used the Ysio digital X-ray Unit (Siemens Healthcare GmbH, Erlangen, Germany) for the image acquisition protocol. Radiographs were taken with the patient barefoot, with the feet facing directly forward, and with a standard foot distance of eight centimetres (20 cm for valgus alignment greater than ten degrees). The X-ray source (automatic exposure control was used to adjust the exposure for each image) was placed perpendicular to the detector at a distance of two metres, and a three-piece cassette was placed behind the patient. Digital radiographs of the hip, knee, and ankle were then taken and electronically “stitched” into a single image with appropriate optical density adjustments. The images were archived in the Picture Archiving and Communication System (PACS, Siemens Healthcare GmbH, Erlangen, Germany) server in the international standard DICOM (Digital Imaging and Communications in Medicine) format [34,35].

Measurements on LLRs studies were performed using Syngo^®^ FastView 2.1 software (Siemens Healthcare GmbH, Erlangen, Germany), a standalone viewer for DICOM images on a Windows^®^ personal computer with a standard display resolution (1920 × 1080 pixels; 16:9). LLRs were assessed by the same senior observer with more than 25 years of experience in angular measurements of the lower extremities. The alignment was measured, and the value obtained was rounded to 0.5 degrees. The mechanical hip–knee–ankle angle (mHKA angle) was obtained by measuring the intersection of the femoral and tibial mechanical axes. The mechanical lateral distal femoral angle (LDFA) was defined as the lateral angle between the femur’s mechanical axis and the distal femur’s joint line (tangent line among the most distal points of the femoral condyles). The mechanical medial proximal tibial angle (MPTA) was defined as the medial angle between the tibia’s mechanical axis and the proximal tibia’s joint line (the line among the deepest points on the medial and lateral tibial condyles) [1,35]. 

The same senior author repeated the blinded measurements six weeks later in a random sample of 150 LLRs. The intraclass correlation coefficient (ICC (2,1); two-way random effects, absolute agreement, single rater) was almost perfect for the mHKA angle (0.986; 95% CI 0.983–0.99), the LDFA (0.974; 95% CI 0.965–0.978), and the MTPA (0.977; 95% CI 0.968–0.98), indicating low intra-observer variability among LLRs over time-spaced measurements. Appropriate intra- and inter-observer correlations, with adequate repeatability coefficients in both pre- and postoperative LLR measurements, have been previously demonstrated [36,37].

The arithmetic hip–knee–ankle angle (aHKA angle) was determined as described by Griffiths-Jones et al. in a previous publication [38], applying the following algorithm: aHKA = MPTA − LDFA. The joint line obliquity (JLO) was calculated by applying the following algorithm: JLO = MPTA + LDFA [1]. 

Statistical analysis was performed using the Statistical Package for the Social Sciences (SPSS), version 25 for Windows (SPSS, Inc., Chicago, IL, USA). Scatterplots were created to describe the proportions of arthritic knees classified based on the CPAK system. Our study followed the ethical standards of the World Medical Association Declaration of Helsinki, as revised in 2013. The institutional review board of the author’s institution approved the study protocol (CEIC-HGURS 17-2024). Given the research’s retrospective nature and medical imaging’s anonymisation, this study was considered exempt from requiring patients’ informed consent.

## 3. Results

Table 1 shows patient characteristics after the removal of outliers. The mean LFDA was 89.8° ± 2.82°, and the mean MPTA was 86.4° ± 2.77°. The mean mHKA and aHKA angles were 172.28° ± 6.22° and −3.4° ± 4.3°, respectively.

Based on the aHKA result, 59.1% of the cases were varus (less than −2°), 32.7% were neutral (0° ± 2°), and 8.2% were valgus (greater than +2°). Based on the JLO result, 56.7% of the cases had a distal apex (less than 177°), 39.9% had a neutral apex (180° ± 3°), and 3.4% had a proximal apex (greater than 183°).

**Figure 1:**
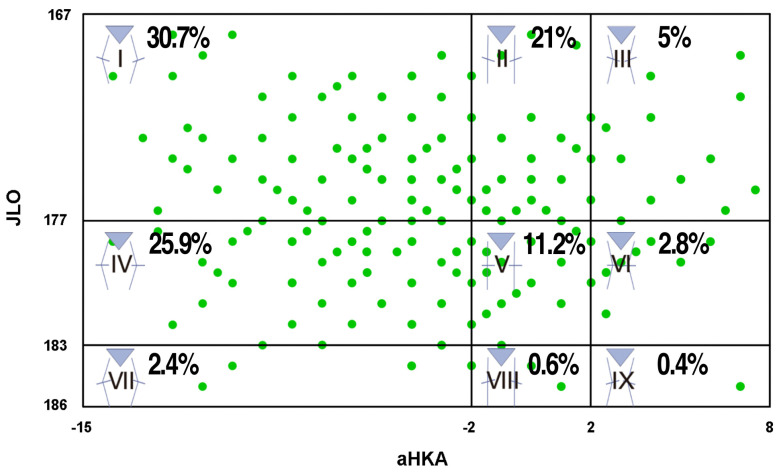
CPAK classification for the studied population.

The CPAK classification is shown in Figure 1 and Table 2. In total, 40% of bilateral cases in both knees matched the type of CPAK, and 60% did not. As part of our data cleaning process, we have identified the values below Q1− (1.5 interquartile range [IQR]) and above Q3+ (1.5 IQR). These values (27 out of 528; 5.11%), which we consider outliers, have been removed from each distribution. This step is crucial, as it avoids including patients with significant arthritic bone loss, measuring errors (for instance, due to flexion contracture on digital LLRs), or data entry errors in the database. 

The above plots aHKA angles against JLO for the arthritic population of our series (after the removal of outliers), showing the distribution by percentage in the nine CPAK types. The data ranges included in each group were as follows: I, IV, VII: aHKA [−16°, −2°]; II, V, VIII: aHKA [−2°, 2°]; III, VI, IX: aHKA [2°, 8°]; I, II, III: JLO [167°, 177°]; IV, V, VI: JLO [177°, 183°]; VII, VIII, IX: JLO [183°, 186°]. aHKA, arithmetic hip–knee–ankle angle; JLO, joint line obliquity.

After removing the outliers, the most common CPAK distribution in our Spanish southeast OA population was type I (30.7%), followed by type IV (25.9%), type II (21%), type V (11.2%), type III (5%), type VI (2.8%), type VII (2.4%), type VIII (0.6%), and type IX (0.4%).

**Figure 2:**
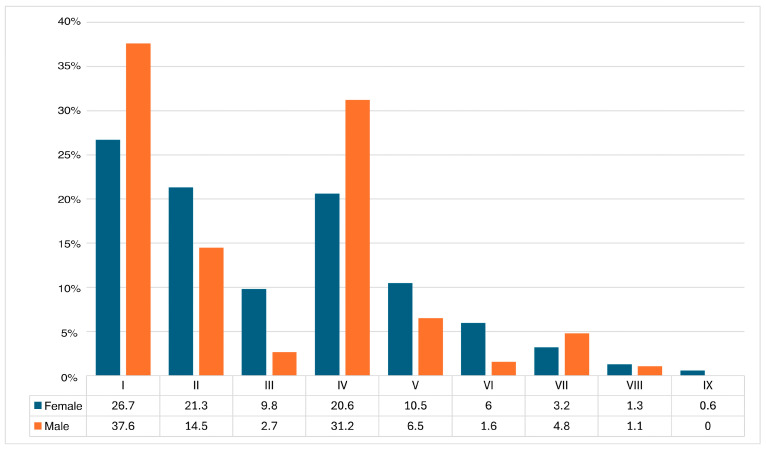
Gender distribution by CPAK classification for the studied population.

We have contrasted the percentage distribution by gender and CPAK type. For this analysis, we have also removed outliers outside of the IQR. As the bar graph in Figure 2 shows, there is a greater tendency for females to be in the neutral and valgus limb alignment groups. Using the Mann–Whitney–Wilcoxon test as a non-parametric method to analyse the differences between the percentages of cases in each theoretical knee type, the *p*-value is 0.73, concluding that there are no statistically significant differences between the males and females in the distribution of cases in the different types of CPAK classification in the population sample studied. We matched the number of male and female cases to assess how this affects the CPAK classification. After matching the groups, the most common CPAK distribution was type I (35.2%), followed by type IV (26.1%), type II (18.8%), type V (9%), type VII (3.8%), type III (3.5%), type VI (1.8%), type VIII (1.3%), and type IX (0.5%).

The above is the gender distribution by CPAK classification for the studied population after removing outliers. The values are in percentages. I to IX are CPAK types.

## 4. Discussion

Our study aimed to describe the CPAK distributions in a Spanish southeast OA cohort. To our knowledge, this is the first study to elucidate the knee phenotype of arthritic Spanish subjects according to the CPAK classification system.

The CPAK classification reported by MacDessi et al. in 2021 provides a simple and pragmatic distribution of nine phenotypes for coronal knee alignment based on constitutional limb alignment and joint line obliquity that can be used in healthy and arthritic knees [1]. The CPAK classification is not the only classification proposed for the coronal plane of the knee [20,21,39,40]. In 2018, Lin et al. classified the alignment of the lower extremities into 5 types (of 27 possible phenotypes) based on the mechanical lateral distal femoral angle and the mechanical medial proximal tibial angle. The authors postulated that this classification might allow for quick and easy interpretations of femoral and tibial coronal alignment and guidance for the preoperative planning of TKA [40]. In 2019, Hirschmann et al. introduced the concept of the functional knee phenotype, with 125 possible phenotypes, 43 of which were considered clinically relevant [20,39]. 

Various authors have also criticised the CPAK classification. For example, Şahbat et al. state that the calculation of the JLO in the CPAK classification may be misleading when defining the position of the apex of the knee joint line obliquity or knee joint line orientation angle (the angle formed by the line parallel to the ground and the line tangential to the tibial condyles), as the agreement between them is less than 50% in their study [41]. Loddo et al. affirm that the CPAK classification may not fully address segmental coronal extra-articular knee deformities and that the CPAK matrix groups do not show a direct correlation with a specific extra-articular deformity pattern [42]. Despite criticism, the CPAK classification is widely used to define the coronal plane’s different morphotypes, providing a common language among healthcare professionals. The advantages of the CPAK classification have been postulated as its excellent inter-rater reliability [1], its exclusive use of LLRs, its simple measurement protocol, and its worldwide use. It helps assess which types benefit most from one alignment philosophy or another [1,26,43,44,45]. Some studies have demonstrated the effectiveness of the CPAK classification in estimating the constitutional alignment of the lower limb following OA [38,43]. Some research even suggests that pre-diseased coronal alignment can be predicted accurately [46]. This information is beneficial for individual or physiological alignment options in TKA surgery. Not all studies agree on the CPAK classification’s stability for healthy and arthritic knees. Some investigations postulate changes in the distribution of phenotypes with OA progression [28,30]. We know that the aHKA and JLO significantly decreased with OA progression [47] and that there is a tendency to have higher rates of varus alignment in Kellgren–Lawrence grades 3 or 4 versus more incipient OA stages (grade 1 and 2) [29]. However, the distributions of the CPAK phenotypes were similar before and after OA development [47]. MacDessi et al. [1] reported similar distributions of all CPAK phenotypes, concluding that the CPAK classification system is helpful for healthy and arthritic knees.

We are beginning to understand more about the phenotypic differences in the knee between populations from different geographical and ethnic backgrounds (Table 3). Compared to the Caucasian OA population, the CPAK distributions in the Asian OA population were very similar. More than half belonged to CPAK type I, followed by types II, IV, III, and V [48]. Coetzze et al. recently published their first study on an African population. The authors investigated OA patients from a single institution in South Africa, and they found a divergence of CPAK phenotypic knee patterns relative to other international studies, with much higher proportions of valgus phenotypes (3 and 6), as shown in Table 3 [47]. The wide geographical variation in the prevalence of CPAK types between healthy subjects and arthritic patients may be invaluable in defining different individualised alignment strategies for TKA surgery. In compliance with the results obtained in our research, the OA population studied has a percentage distribution according to the CPAK classification, like the OA populations described by Sappey-Marinier et al. (France) [27], Tarassoli et al. (Australia) [24], and Şenel et al. (Turkey) [47], but quite different from the rest of the descriptions, as shown in Table 3. 

We observed a higher percentage of CPAK type IV (25.9%) than in any previously published studies (as Table 3 shows). Consistent with published studies in different geographical regions and with different ethnicities, the percentage of CPAK type V patients (the postoperative phenotype targeted by the mechanical alignment philosophy in TKA surgery) is low: in our particular case, it was 11.2%. In our research, the analysis of the gender distribution coincides with the tendency of the female sex to cluster in the neutral and valgus types, as in the reports of Huber et al. [51] and Steele et al. [29].

Despite new alignment proposals, the systematic option of MA [7] and the measured resection technique for the femoral component rotational placement [52,53] remain the gold standard and the most widely used options. Dissatisfaction with knee replacement surgery has been reported in a variable percentage (between 10% and 20%) [54,55]. However, we ignore how the achieved postoperative alignment influences this percentage. One of the hypotheses is the change in the native orientation of the joint line and, consequently, the change in ligament balancing. The MA targets a neutral postoperative coronal axis by placing components perpendicular to the mechanical axis of the femur and the tibia; this aims to achieve a CPAK type V phenotype for all patients [23]. However, this ignores knee alignment differences, morphology, and biomechanics variations. For populations with a low prevalence of CPAK type V (11.2% in our research), applying an MA approach to all patients may not be favourable [23]. When performing TKA with MA, most patients had a postoperative neutral JLO, and this change in the apex may impact dissatisfaction. Many studies [56,57,58,59] have shown improved clinical outcomes by maintaining postoperative varus alignment in patients with preoperative varus alignment. These studies support that restoring preoperative knee alignment would improve postoperative outcomes [27,44]. Therefore, it seems essential to have preoperative references (such as the CPAK classification, although limited to the coronal plane) to restore each patient’s constitutional alignment and to minimise the degree of dissatisfaction with TKA surgery outcomes.

There were several potential limitations in this study. First, we have only classified the cases according to the CPAK classification. We have not contrasted the cases with the functional knee phenotype concept [21]. Second, this was a retrospective cohort study based on the database of a single institute. Although our study reached an adequate number of cases, its limitation as a single-centre study of patients originating from a determined region of our country limits the generalisability of the results to the whole population. Conducting studies with the participation of multiple centres from different geographical regions of our country may provide results that more accurately reflect the Spanish population. Third, this study only included data from patients with knee OA (Kellgren–Lawrence grade 3 or 4) and did not examine healthy patients. Long-term follow up of healthy individuals would be desirable to identify changes in lower limb alignment over time. Despite analysing only OA patients, statistical methods eliminated any outliers, allowing both errors and cases with severe deformity due to osteoarthritis to be avoided. Fourth, the OA group studied did not have an equal gender distribution, with the number of females being 1.8 times higher than that of males, typical of a TKA population. Fifth, we removed values outside the interquartile range, as we considered these values to be outliers in the dataset and to not reflect the natural variability of the data. However, this would likely have a limited effect on the results, given the large numbers in this cohort. Sixth, a single observer made all of the measurements, so we lack information on inter-observer variability. Despite the abovementioned concerns, our findings may contribute information on the most frequent OA phenotype in the southeast Spanish population according to the CPAK classification. In our group, this was essential, as we performed a large volume of surgeries following the philosophy of unrestricted kinematic alignment, and it is of enormous value to identify the phenotypes in which there is a greater likelihood of optimal balance (I, II, and IV) [1,44].

## 5. Conclusions

We have described the distribution according to the CPAK classification in a sample of the OA population from southeastern Spain. We compared the percentage distribution of each type with publications from other regions and other ethnic groups and found differences (mainly in Asian populations). The percentage of type IV in the population studied is higher than those previously reported by other authors. In our sample, more than 75% of the patients were classified as type I, II, and IV. Despite possible biases, our study’s results provide relevant information on the distribution of phenotypes in the coronal plane that can help to make decisions regarding the most appropriate alignments, for example.

## Figures and Tables

**Table 1:** Patient characteristics.

n	501 Knees
Number of patients	447
Female/male	288 (64.4%)/159 (35.6%)
Left side/Right side	236 (47.1%)/265 (52.9%)
Age	69.91 ± 6.29 years (56–83 years)
Height	160.49 ± 8.92 cm (140–187 cm)
Weight	76.45 ± 9.63 kg (52.5–106 kg)
BMI	29.79 ± 3.9 kg/m^2^ (20–41.79 kg/m^2^)
mHKA	172.28° ± 6.22° (153°–194°)
LDFA	89.8° ± 2.82° (81°–98°)
MPTA	86.4° ± 2.77° (78°–96°)
aHKA	−3.4° ± 4.3° (−14°–7.5°)
JLO	176.2° ± 3.58° (168°–188°)

Abbreviations: BMI, body mass index; mHKA, mechanical hip–knee–ankle angle; LDFA, lateral distal femoral angle; MPTA, medial proximal tibial angle; aHKA, arithmetic hip–knee–ankle; JLO, joint line obliquity.

**Table 2:** Percentage distribution according to the CPAK classification.

CPAK Type	Without Outliers (*n* = 501)
CPAK I (varus and apex distal)	30.7%
CPAK II (neutral and apex distal)	21%
CPAK III (valgus and apex distal)	5%
CPAK IV (varus and apex neutral)	25.9%
CPAK V (neutral and apex neutral)	11.2%
CPAK VI (valgus and apex neutral)	2.8%
CPAK VII (varus and apex proximal)	2.4%
CPAK VIII (neutral and apex proximal)	0.6%
CPAK IX (valgus and apex proximal)	0.4%

**Table 3:** Percentage distribution according to the CPAK classification of arthritic populations.

CPAK Type	MacDessi et al. [1] (*n* = 500) Australia	MacDessi et al. [1] (*n* = 138) Australia	Sappey-Marinier et al. [27] (*n* = 1078) France	Tarassoli et al. [24] (*n* = 88) Australia	Mulpur et al. [28] (*n* = 250) India	Toyooka et al. [25] (*n* = 500) Japan	Nomoto et al. [45] (*n* = 60) Japan	Şenel et al. [47] (*n* = 408) Turkey	Yang et al. [30] (*n* = 500) Korea	Gao et al. [48] (*n* = 477) China	Liu et al. [49] (*n* = 434) China	Palanisamy et al. [31] (*n* = 352) India	Coetzee et al. [50] (*n* = 344) South Africa	León et al. 2024 (*n* = 501) Spain
CPAK I	19.4%	16%	33.4%	33%	58.8%	53.8%	68.3%	28.2%	53.8%	43.6%	53.9%	56.5%	15.5%	30.7%
CPAK II	32.2%	38%	19.5%	27.3%	13.8%	25.4%	21.7%	31.6%	17.6%	21.6%	17.1%	14.5%	25.5%	21%
CPAK III	15.4%	20%	10.6%	15.9%	1.4%	8.2%	6.7%	13.5%	1.6%	10.5%	9.2%	5.7%	28.6%	5%
CPAK IV	9.8%	10.8%	10.2%	3.4%	18.2%	7.2%	3.3%	10.3%	17.4%	11.5%	12.7%	13.6%	7.4%	25.9%
CPAK V	14.6%	8.6%	18.9%	5.7%	3.4%	4.4%	0%	12.3%	7.2%	7.5%	2.3%	5.7%	8.6%	11.2%
CPAK VI	7.4%	5%	6.3%	12.5%	1%	1%	0%	2.5%	0.6%	3.8%	3%	0.6%	16.2%	2.8%
CPAK VII	0.6%	0%	0.4%	0%	2.8%	0%	0%	1%	2%	1.1%	0.9%	2%	0.5%	2.4%
CPAK VIII	1.6%	0%	0.6%	1.1%	0.6%	0%	0%	0%		0%	0.2%	0.9%	0.2%	0.6%
CPAK IX	0.4%	0%	0.1%	1.1%	0%	0%	0%	0.7%		1.4%	0.7%	0.6%	0.7%	0.4%

## Data Availability

The datasets used and/or analysed during the current study are available from the corresponding author on reasonable request.
